# Adjunct ketamine treatment of depression in treatment‐resistant schizophrenia patients is unsatisfactory in pilot and secondary follow‐up studies

**DOI:** 10.1002/brb3.1600

**Published:** 2020-03-15

**Authors:** Chuanjun Zhuo, Xiaodong Lin, Hongjun Tian, Sha Liu, Haiman Bian, Ce Chen

**Affiliations:** ^1^ Department of Psychiatry School of Mental Health Jining Medical University Jining China; ^2^ Department of Psychiatric‐Neuroimaging‐Genetics Laboratory (PNG_Lab) Wenzhou Seventh People's Hospital Wenzhou China; ^3^ PNGC‐Lab Tianjin Mental Health Centre Tianjin Anding Hospital Tianjin China; ^4^ Department of Psychiatry First Hospital of Shanxi Medical University Tainyuan China; ^5^ Department of Radiology The Fourth Centre Hospital of Tianjin Tianjin Medical University Affiliated Fourth Centre Hospital Tianijn China

**Keywords:** depressive symptoms, fMRI, ketamine, regional homogeneity, schizophrenia

## Abstract

**Objective:**

To investigate the effects of adjunct ketamine treatment on chronic treatment‐resistant schizophrenia patients with treatment‐resistant depressive symptoms (CTRS‐TRD patients), including alterations in brain function.

**Methods:**

Intravenous ketamine (0.5 mg/kg body weight) was administered to CTRS‐TRD patients over a 1‐hr period on days 1, 4, 7, 10, 13, 16, 19, 22, and 25 of our initial pilot study. This treatment method was subsequently repeated 58 days after the start of the pilot study for a secondary follow‐up study. Calgary Depression Scale for Schizophrenia (CDSS), Positive and Negative Syndrome Scale (PANSS), and regional homogeneity (ReHo) results were used to assess treatment effects and alterations in brain function throughout the entire duration of our studies.

**Results:**

Between day 7 and day 14 of the first treatment, CDSS scores were reduced by 63.8% and PANSS scores were reduced by 30.04%. In addition, ReHo values increased in the frontal, temporal, and parietal lobes. However, by day 21, depressive symptoms relapsed. During the second treatment period, CDSS and PANSS scores exhibited no significant differences compared to baseline between day 58 and day 86. On day 65, ReHo values were higher in the temporal, frontal, and parietal lobes. However, on day 79, the increase in ReHo values completely disappeared.

**Conclusions:**

Depressive symptoms in CTRS‐TRD patients were alleviated with adjunct ketamine treatment for only 1 week during the first treatment period. Moreover, after 1 month, the antidepressant effects of ketamine on CTRS‐TRD patients completely disappeared. Correspondingly, ReHo alterations induced by ketamine in the CTRS‐TRD patients were not maintained for more than 3 weeks. These pilot findings indicate that adjunct ketamine treatment is not satisfactory for CTRS‐TRD patients.

## Graphical abstract

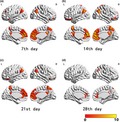

## 1 INTRODUCTION

Approximately 20% of chronic schizophrenia patients experience moderate‐to‐severe depressive symptoms (Conley, Ascher‐Svanum, Zhu, Faries, & Kinon, 2007; Upthegrove et al., 2010). Prior models have suggested that there is a dichotomy between schizophrenia and depression (Craddock & Owen, 2010). However, recent evidence suggests that depressive systems may predict poorer outcomes in schizophrenia (Gardsjord et al., 2016; Upthegrove et al., 2010). Moreover, depressive symptoms have been linked to suicidality (Dutta et al., 2011), poor functional recovery, and poor quality of life in schizophrenic patients (Conley et al., 2007; Dutta et al., 2011). Use of antidepressant drugs to treat depressive symptoms in schizophrenic patients taking antipsychotics is reported to have poor efficacy (Gregory et al., 2017; Helfer et al., 2016; Tiihonenl et al., 2016). Therefore, researchers have undertaken the development of animal models to explore possible depression treatments for schizophrenic patients (Samsom & Wong, 2015).

Ketamine is an effective antidepressant agent, especially in patients with treatment‐resistant depressive (TRD) symptoms (Bobo et al., 2016; Krystal et al., 2019; Phillips et al., 2019; Silberner, 2019). In healthy adults, a single administration of ketamine (0.5 mg/kg) can induce immediate psychotomimetic symptoms, which recede within 2 hr (Krystal et al., 2019). The antidepressant effects of ketamine have been associated with alterations in brain functional activity, mainly in the medial prefrontal cortex (mPFC), anterior cingulate cortex (ACC), posterior cingulate cortex, precuneus, angular gyrus, orbitofrontal cortex (OFC), subgenual anterior cingulate cortex, superior temporal gyrus, middle temporal gyrus, and hippocampus (Abdallah, Averill, et al., 2017; Carlson et al., 2013; Evans et al., 2018; Gartner et al., 2019; Li et al., 2018; Reed et al., 2018). These previous studies provide important information to guide further investigations of mechanisms mediating the antidepressant effects of ketamine. They also provide insights regarding optimization of ketamine administration and they expand the spectrum of diseases for which ketamine treatment may be applicable based on TRD symptoms.

Atypical brain activity findings have been reported for both schizophrenia (Dezhina, Ranlund, Kyriakopoulos, Williams, & Dima, 2019; Glausier & Lewis, 2018; Krajcovic & Fajnerova, 2019) and major depressive disorder (Arnone, 2019; Marwood et al., 2018; Sankar et al., 2018). Local temporal homogeneity of regional blood oxygen level‐dependent signals, referred to as ReHo, has been used to assess resting‐state neural activity (Chen et al., 2013; Wei et al., 2017, 2018; Xia et al., 2019; Yang et al., 2019; Zang et al., 2004). Moreover, since ReHo focuses on similarities over time, it can also be used to assess functional brain alterations (Chen et al., 2013; Paakki et al., 2010). Interestingly, similarities have been identified between schizophrenia‐ and major depressive disorder‐related brain alteration patterns, particularly in regard to ReHo data. Furthermore, similar antidepressant and antipsychotic brain activity normalization effects have been reported for these two patient populations, most notably in the temporal, parietal, and frontal lobes (Abbott, Jaramillo, Wilcox, & Hamilton, 2013; Arnone, 2019; Kraus et al., 2017; Lesh et al., 2015). Interestingly, low‐dosage ketamine does not appear to deteriorate psychotic symptoms in patients with schizophrenia, and it does not induce psychotic symptoms in depression patients or in patients with bipolar or post trauma stress disorders (Lener et al., 2017; Liriano et al., 2019; Molero et al., 2018).

Considering the aforementioned findings, we decided to investigate the effects of combining ketamine with therapeutic agents on TRD symptoms and brain ReHo in chronic treatment‐resistant schizophrenia (CTRS) patients. We hypothesized that augmentation with a ketamine treatment regimen would improve TRD symptoms in CTRS patients, and such effects would be accompanied by alterations in pivotal regions of the brain.

## 2 METHODS

### 2.1 Patients

This study was approved by local institutional review boards. Inclusion criteria were as follows: (a) a diagnosis of CTRS, as described by Howes (Nierenberg & Amsterdam, 1990); (b) comorbid TRD symptoms, according to Nierenberg's criteria (Akil et al., 2018; Grover et al., 2017); (c) active disorder presentation; (d) an intelligence quotient ≥80; and (e) willingness of the patient (and guardian when appropriate) to participate in this study. Exclusion criteria were as follows: (a) moderate‐to‐severe physical disease (e.g., respiratory, cardiovascular, endocrine, neurological, liver, or kidney disease) comorbidity; (b) personal or family history of substance abuse; (c) current nicotine addiction; (d) currently receiving electroconvulsive therapy; (e) loss of consciousness for more than 5 min due to any cause; (f) left‐handedness, as determined with the Annett Hand Preference Questionnaire; and (g) any magnetic resonance imaging (MRI) contraindication, including claustrophobia. According to these strict enrollment criteria, a total of 15 patients were eligible to participate in this study. All of them and their guardians provided written informed consent.

### 2.2 Adjunct ketamine administration methods

Following a baseline assessment of depressive and psychotic symptoms, medication dosages were standardized during a 4‐week adjunct ketamine treatment period. Briefly, intravenous ketamine (0.5 mg/kg body weight) was administered over a 1‐hr period on days 1, 4, 7 ,10, 13, 16, 19, 22, and 25 of the study starting at 6 p.m. Heart rhythm, blood pressure, and blood oxygen were monitored during, and up to 2 hr after, the infusion of ketamine. Liver and renal function were tested twice a week. Heart rhythm, blood pressure, and blood oxygen were monitored from 6 p.m. to 8 p.m. for totally 2 hr during the period of ketamine administration and thereafter. Physical signs and patient‐reported symptoms were also noted during this monitoring period. Adjunct ketamine treatment was immediately discontinued if a patient exhibited any adverse secondary effects (ASEs), which were considered high risk by the patient's neurologist or cardiologist.

### 2.3 Main and secondary effect assessments

The Calgary Depression Scale for Schizophrenia (CDSS) and Positive and Negative Syndrome Scale (PANSS) were used to assess depressive and psychotic symptoms once a week. To detect ASE emergence, monitoring indices, consults with neurologists and cardiologists, and the Treatment Emergent Symptom Scale (Zhang, 1990) were used.

### 2.4 Acquisition of brain MRI data

Functional MRI (fMRI) data were collected at five time points relative to the initiation of adjunct ketamine treatment: at baseline (pretreatment) and then on days 7, 14, 21, and 28. The fMRI examinations were performed with a 3.0‐T Discovery MR750 system (GE). Briefly, each participant was instructed to lie still while staying awake with a relaxed mind during scanning. Each participant was fitted with foam padding and earplugs to limit head motion and the effects of external noises. A single‐shot echo‐planar sequence for resting‐state fMRI was applied as follows: repetition/echo times = 2,000/45 ms, field of view = 220 mm^2^, matrix = 64 × 64, flip angle = 90°, slice thickness = 4 mm, and gap = 0.5 mm. Each functional run consisted of 180 image volumes over a 32‐axial‐slice brain volume for each patient. T1‐weighted three‐dimensional images (188 slices) were obtained with a brain volume sequence constituted by the following parameters: repetition/echo/inversion times = 8.17/3.18/450 ms, field of view = 256 mm^2^, matrix = 256 × 256, and slice thickness = 1 mm.

The fMRI data were preprocessed by using SPM8 and DPARSF V2.3 programs. The first ten images were excluded from each patient's scan dataset to allow signal equilibration. Slice timing was performed to correct for interslice temporal differences. Head motion was screened and corrected for by using the rigid body realignment method (Zhang et al., 2016).

### 2.5 Statistical analysis

Before versus after treatment, differences in ReHo were subjected to family‐wise error correction. A paired *t* test was used to compare CDSS and PANSS scores to ketamine treatment‐induced changes in ReHo. *p*‐values < .05 were considered statistically significant.

### 2.6 Image data preprocessing

Pretreatment and posttreatment fMRI datasets were preprocessed separately by using three programs: FMRIB Software Library, version 5 (http://fmrib.ox.ac.uk/fsl), Analysis of Functional NeuroImages (http://afni.nimh.nih.gov/afni/), and FreeSurfer, version 5.3 (http://surfer.nmr.mgh.harvard.edu/). High‐resolution T1 images aligned to the cortical surface of each patient were reconstructed in accordance with the FreeSurfer pipeline. Briefly, after registering the images to the Talairach atlas and applying a bias‐field correction, skull stripping, intensity normalization, surface modeling, and spherical mapping were conducted. Subsequently, slice timing correction, deobliquing, and motion correction processes were applied to the data. Whole images were normalized according to their mean intensity values and then were scaled 10,000 times. Both linear and quadratic trends were removed from the signals. A transformation matrix generated by boundary‐based registration was applied to coregister each image with T1 images. A principal component analysis of the time course to regress out five major components of white matter and cerebrospinal fluid was subsequently performed, thereby reducing physiological (and other) noise.

### 2.7 ReHo estimation and analysis

Regional homogeneity analysis was performed to investigate spontaneous neuronal activity and short‐range connectivity, without the need for an a priori hypothesis (Zang et al., 2004). Briefly, regional similarity across the time series was determined by calculating Kendall's coefficient of concordance of target‐region surrounding voxels (*n* = 26) for each target voxel. Preprocessed fMRI data were subjected to low band pass filtering (0.009–0.1 Hz) and then were resampled as 3‐mm isotropic voxels without spatial smoothing. Voxel ranks were computed at each repetition time. ReHo values were calculated along the middle of the gray matter–white matter boundary and projected to surface vertices. Surface alignment of functional signals can reduce interindividual variability related to cortical folding and limit activation spread over distant regions in spatial smoothing processes. Surface fMRI data obtained before and after ketamine treatment were subjected to pair‐wise registration. Surface data were moved to a common spherical surface and then were smoothed spatially with a 5‐mm full‐width at half‐maximum Gaussian kernel. The ReHo values obtained were transformed into *Z* scores in the surface model, and these were used in a group‐level statistical analysis.

To assess drug treatment effects, general linear modeling (with participant age and intelligence quotient controlled for) was applied. Criteria for identifying significant clusters were as follows: cluster *p* < .0001; cluster size > 10 voxels after 10,000 Monte‐Carlo *z* statistic simulations; and cluster *p* < .05 after two‐tailed test and correction for hemispheric tests.

To identify potential cluster‐wise ReHo change relationships with changes in symptoms (which initially varied across participants), a correlation analysis between percent changes in ReHo values and symptom presentation measures was performed. Spearman's rank‐order method was used for correlation analysis (*N* = 12 participants), which was conducted by using in‐house code written in MATLAB (MathWorks, Inc.). Changes in ReHo values were subjected to family‐wise error correction.

## 3 RESULTS

### 3.1 Demographic and clinical characteristics of the analyzed cohort

All 15 enrolled CTRS patients with TRD symptoms (CTRS‐TRD patients) completed the adjunct ketamine treatment (0% drop‐out). However, complete fMRI data could not be obtained for three of the 15 enrolled participants. Therefore, our final analysis only included data from 12 participants. Demographic and clinical characteristics of this final cohort are summarized in Table 1. None of the participants complained of ketamine‐induced ASEs, although one patient reported experiencing visual hallucinations. The latter patient reported seven hallucinations within 30 min after the first ketamine infusion. The hallucinations were of objects (e.g., an apple and an eggplant), and the longest duration for an individual hallucination was 2 min.

**Table 1:** Mean demographic and clinical characteristics of CTRS‐TRD patients undergoing augmentation ketamine treatment in our initial pilot study (*N* = 12)

Variable	Before treatment	After 2 weeks of treatment	After 4 weeks of treatment	*F*	*p*‐value
Age, years	35.16 ± 7.63	–	–	–	–
Education, years	16.62 ± 3.96	–	–	–	–
Illness duration, years	5.38 ± 1.42	–	–	–	–
Male/female ratio	7/5	–	–	–	–
Chlorpromazine equivalent dose	1,250.70 ± 200.80	–	–	–	–
CDSS score	16.50 ± 3.94	5.98 ± 1.94	14.28 ± 2.30	14.298	<.001
PANSS scores					
Total	83.90 ± 9.23	73.04 ± 10.10	80.23 ± 8.51	3.051	.016
Positive	25.60 ± 3.75	25.44 ± 4.05	26.11 ± 5.14	0.198	.921
Negative	27.71 ± 5.19	27.31 ± 4.93	26.00 ± 5.36	0.194	.848
General psychopathological symptoms	29.90 ± 5.41	20.81 ± 4.97	26.00 ± 3.85	4.286	<.001
TESS score	22.57 ± 6.55	21.54 ± 5.33	20.99 ± 4.70	0.688	.433

Note

Mean values are reported ± standard deviation.

Abbreviations: CDSS, Calgary Depression Scale for Schizophrenia; CTRS‐TRD, chronic treatment‐resistant schizophrenia with treatment‐resistant depressive symptoms; PANSS, Positive and Negative Syndrome Scale; TESS, Treatment Emergent Symptom Scale.

### 3.2 Treatment effects of ketamine

**Figure 1:**
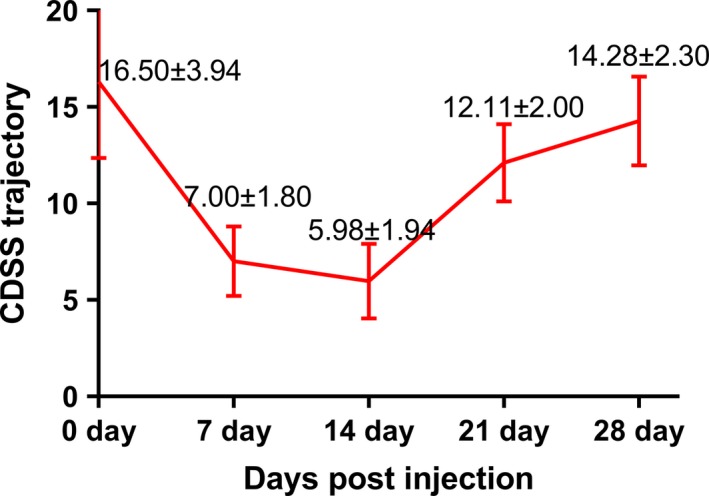
CDSS scores at each assessment timepoint

Adjunct ketamine (0.5 mg/kg, intravenous [i.v.] over 1 hr) was found to reduce both CDSS (depressive symptoms) scores (63.7% decrease) and PANSS general psychopathological symptom scores (30.04% decrease) significantly between days 7 and 14 after the first ketamine treatment (Table 1). The mean CDSS score for the cohort subsequently increased between days 14 and 21. Then by day 28, the mean CDSS score increased to a level that was statistically similar to the mean CDSS score at baseline, despite maintenance of a fixed ketamine treatment strategy. Trajectory of the change in CDSS scores is shown in Figure 1. Meanwhile, PANSS negative (0.23% decrease) and positive (0.06% decrease) scores did not exhibit significant changes between pre‐ and post‐ketamine adjunct treatment time points (Table 1). The latter results indicate that the adjunct ketamine treatment regimen did not induce activation of psychotic symptoms, and none of the patients exhibited or reported ASEs requiring medical intervention.

### 3.3 Alterations in ReHo values

**Figure 2:**
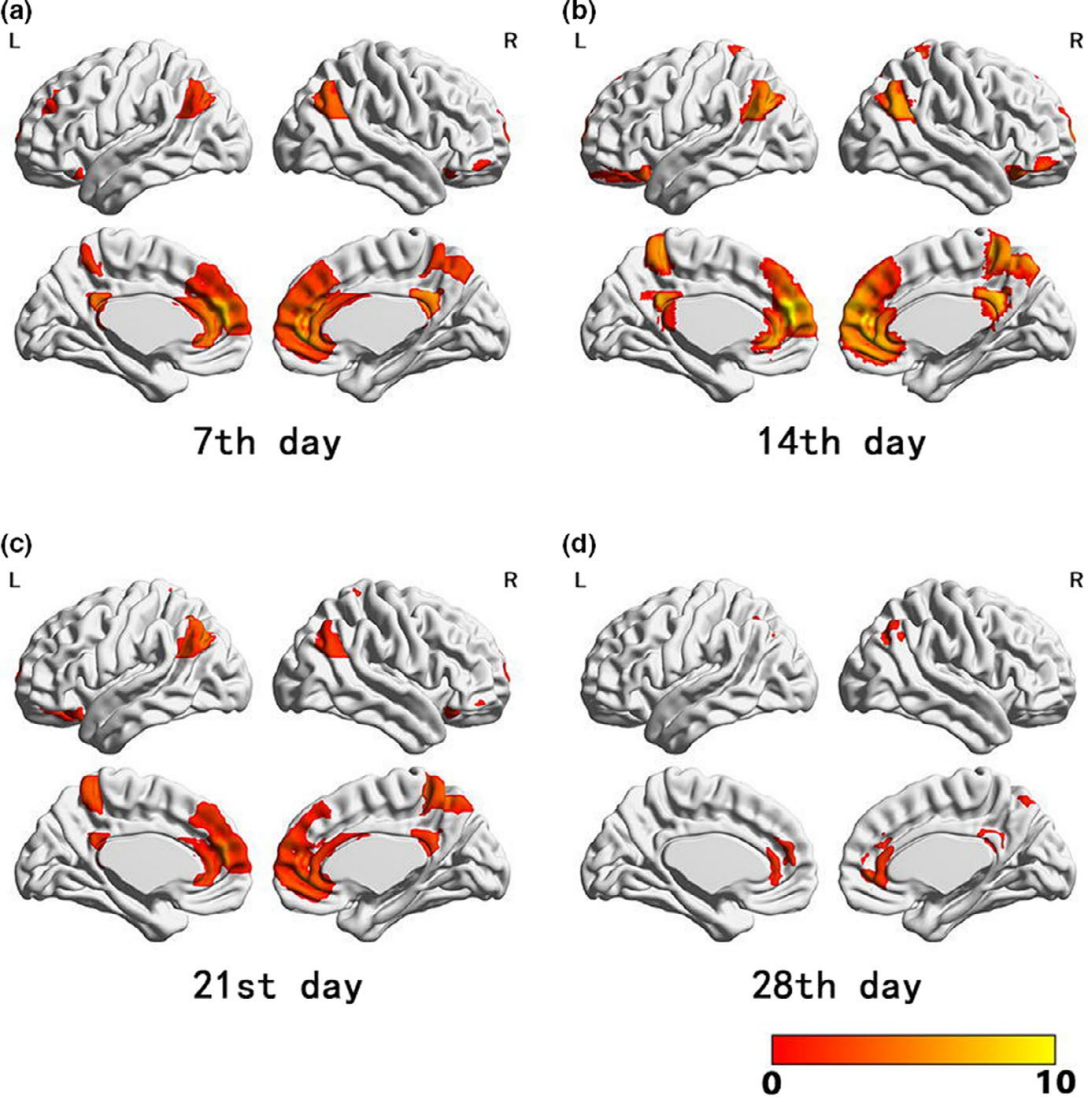
Adjunct ketamine treatment‐induced changes in ReHo values assessed at day 7 (a), day 14 (b), day 21 (c), and day 28 (d) compared with baseline

Compared to pretreatment observations, an increase in ReHo values was observed mainly in the mPFC, ACC, posterior cingulate cortex, precuneus, angular gyrus, and bilateral OFC, beginning from day 7 after the start of ketamine administration (Figure 2a). ReHo values subsequently peaked on day 14 (Figure 2b), they remained high on day 21 (Figure 2c), and then, they exhibited a notable decrease on day 28. The latter level did not differ significantly from baseline (it was unable to withstand family‐wise error correction; Figure 2d). Furthermore, CDSS and PANSS alterations which occurred between days 7 and 14 did not correlate with any regional changes in ReHo.

### 3.4 Secondary follow‐up study

In our pilot study (described above), ketamine augmentation treatment only alleviated depressive symptoms for 1 week. In addition, an increase in ReHo values in the brain was not maintained for more than 3 weeks. We hypothesize that rapid neurotransmitter desensitization weakens a synergistic ketamine effect and is responsible for these results (Bolton, Phillips, & Constantine‐Paton, 2013; Glasgow et al., 2017). Based on these initial findings and the fact that no serious ASEs were observed in our pilot study, we designed a follow‐up study to further investigate the effects of ketamine augmentation treatment on our cohort. To avoid neurotransmitter desensitization, the augmentation treatment protocol was repeated in the same patients 58 days after the start of the pilot study. This time frame was selected so that normalization of neurotransmitter desensitization could occur after completion of the pilot study.

The secondary follow‐up study included all of the original patients, it employed the same method as the pilot study, it was approved by local institutional review boards, and written informed consent was obtained from each patient. During the interval between the end of the pilot study (day 28 from baseline) and the start of the follow‐up study (day 58 from baseline), repetitive transcranial magnetic stimulation (rTMS) was adopted as an aided method. During rTMS treatment, CDSS and PANSS scores did not exhibit significant differences compared to baseline. Then, 1 week before starting the secondary ketamine treatment (day 51), rTMS treatment was discontinued to avoid a confounding factor.

**Figure 3:**
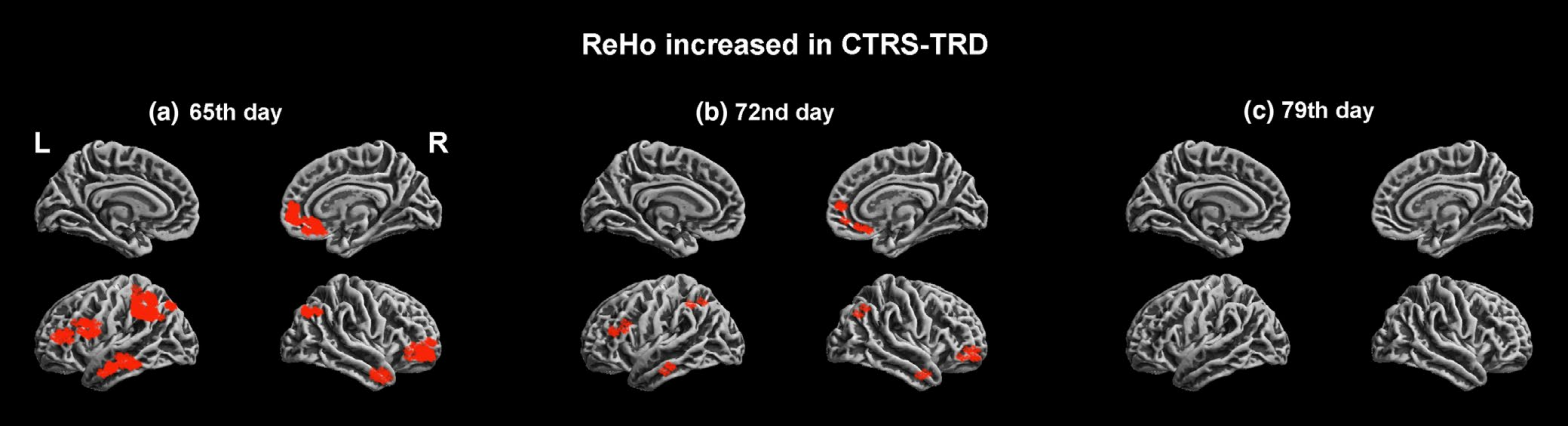
Adjunct ketamine treatment‐induced ReHo changes during the secondary follow‐up study. ReHo values were compared for: day 65 (a), day 72 (b), and day 79 (c) versus day 58 (just prior to the start of augmentation ketamine treatment for the secondary follow‐up study)

Ketamine augmentation treatment in the secondary study was started on day 58 from baseline. Alterations in ReHo values were subsequently observed at days 65, 72, and 79. At day 58 (just before augmentation ketamine treatment was started), fMRI data were acquired and used as the baseline reference for the secondary follow‐up study. After 7 days of ketamine treatment (on day 65), an increase in ReHo values was observed in the temporal, frontal, and parietal lobes compared to that on day 58. However, after further 7 days of ketamine treatment (on day 72), although an increase in Reho values remained observed compared to that on day 58, the ReHo *T*‐value was lower than the *T*‐value observed on day 65. On day 79, the increases in ReHo values had disappeared compared to day 58 (Figure 3).

During the secondary follow‐up study, assessments of clinical symptoms by CDSS and PANSS were performed four times (every 1 week). Compared to baseline, none of the CDSS or PANSS scores exhibited significant differences. There were also no significant differences in CDSS or PANSS scores at the two assessments (days 65 and 72) compared to day 58 (after completion of the pilot study; Table 2). Finally, there were no significant differences in the CDSS and PANSS scores between baseline and day 79 (CDSS: 16.50 ± 3.94 vs.16.12 ± 2.30, 65th day, 72nd day*p* = .800; PANSS: 83.90 ± 9.23 vs. 81.99 ± 8.87, *p* = .555, respectively, in each case). Taken together, these findings indicate that efficacy of the secondary ketamine augmentation treatment was insignificant, despite observed alterations in brain activity.

**Table 2:** Mean demographic and clinical characteristics of CTRS‐TRD patients undergoing augmentation ketamine treatment in our secondary follow‐up study (*N* = 12)

Variable	At day 58 before treatment	At day 65 of treatment	At day 72 of treatment	*F*	*p*‐value
Age, years	35.16 ± 7.63	–	–	–	–
Education, years	16.62 ± 3.96	–	–	–	–
Illness duration, years	5.38 ± 1.42	–	–	–	–
Male/female ratio	7/5	–	–	–	–
Chlorpromazine equivalent dose	1,250.70 ± 200.80	–	–	–	–
CDSS score	15.79 ± 2.20	14.98 ± 1.97	16.12 ± 2.30	0.557	.429
PANSS scores					
Total	82.52 ± 7.58	80.44 ± 7.89	81.99 ± 8.87	0.751	.236
Positive	24.30 ± 1.78	25.17 ± 5.40	25.07 ± 5.14	0.562	.403
Negative	26.10 ± 2.59	26.13 ± 2.97	26.90 ± 5.36	0.147	.888
General psychopathological symptoms	32.12 ± 3.78	29.59 ± 4.02	30.02 ± 5.38	0.373	.607
TESS score	22.57 ± 6.55	21.54 ± 5.33	20.99 ± 4.70	0.122	.877

Note

Mean values are reported ± standard deviation.

Abbreviations: CDSS, Calgary Depression Scale for Schizophrenia; CTRS‐TRD, chronic treatment‐resistant schizophrenia with treatment‐resistant depressive symptoms; PANSS, Positive and Negative Syndrome Scale; TESS, Treatment Emergent Symptom Scale.

## 4 DISCUSSION

To the best of our knowledge, this pilot study and secondary follow‐up study are the first to examine the effects of adjunct ketamine treatment on depressive symptoms in patients with CTRS‐TRD. In the initial pilot study, depressive symptoms were only alleviated for 1 week with adjunct ketamine treatment. Meanwhile, an increase in ReHo values in the patients' brains was not maintained for more than 3 weeks. In contrast, adjunct ketamine treatment was ineffective in the secondary follow‐up study, and ReHo values were also not maintained for more than 3 weeks. Of particular note, the brain regions with increased ReHo values differed between the pilot study and the secondary follow‐up study. The areas of the brain which exhibited an increase in ReHo values were more extensive in the secondary follow‐up study than in the pilot study, yet the strength (*T*‐value) of the ReHo values was lower compared with the pilot study.

Regarding the findings of our pilot study, we postulate that the antidepressant effect of our ketamine augmentation treatment can be explained as follows. First, the mPFC, anterior cingulate, posterior cingulate, precuneus, and angular gyrus (all identified as components of the default mode network [DMN]), as well as the OFC (part of the affective network), are key regions related to mood processing (Ismaylova et al., 2018; Smith et al., 2018; Soares et al., 2017; Timbie & Barbas, 2015). Notably, neural activities in the DMN and OFC have been reported to be markedly reduced in depressed patients (Cheng et al., 2018; Hao et al., 2019; Levenson et al., 2014; Schmaal et al., 2017; Subramaniam et al., 2018; van Eijndhoven et al., 2013). Deficits in the DMN and OFC have also been related to affective and memory processing disturbances in schizophrenic patients (Chakirova et al., 2010; Jackowski et al., 2012; Nakamura et al., 2008; Qiu & Lin, 2019; Reske et al., 2007; Rodriguez et al., 2019; Tendolkar et al., 2004; Zong et al., 2019). In our pilot study, increased ReHo values indicate that ketamine can enhance activity in the DMN and OFC, while reducing depressive symptom severity. These findings are consistent with those published by Reed et al. (2018) which demonstrate that ketamine can normalize brain activity during emotionally valenced attentional processing in depressive subjects. Previous studies have also reported that a single ketamine treatment may alleviate treatment‐resistant depression by normalizing aberrant activity in DMN components and in the frontal cortex (Maltbie et al., 2017; Mueller et al., 2018; Murrough et al., 2015). Thus, the findings of our pilot study support the postulation of previous studies.

The antidepressant effect of ketamine has been related to ketamine‐induced increases in glutamate release (Duman & Shinohara, 2019). However, ketamine has been used to establish an animal model of schizophrenia, which would suggest that ketamine possibly activates psychotic symptoms. In our pilot study, no evidence of ketamine‐induced psychotic symptoms was observed. It may be that such effects require a higher dose of ketamine, considering that mid‐range doses of ketamine are currently used for both animal model induction and psychedelic use. Meanwhile, high doses of ketamine are used for anesthesia. Thus, low doses of ketamine may be antidepressive. Indeed, low‐dose ketamine has been associated with repair of disrupted dendrites in the frontal lobes (Ferenczi et al., 2016). Hence, we postulate that low‐dose ketamine does not trigger negative effects in schizophrenic patients, but rather, exerts positive effects. In this context, it is also interesting to note that the glutamatergic system has been reported to affect the efficacy of antipsychotic medications (Wang et al., 2018). Thus, it has been suggested that pharmacological modulation of NMDA receptor function may reverse abnormal glutamatergic transmission, which is hypothesized to occur in schizophrenia (Parkin et al., 2018).

The findings of our pilot study demonstrate a dissociation between clinical and functional changes in the brain following adjunct ketamine administration. For example, while a clinical effect was attenuated within the first week of treatment, attenuation of brain function was not obvious until the second week of treatment. This dissociation phenomenon raises issues to be addressed in future research. We postulate three possible reasons for this dissociation phenomenon. First, neuronal interactions depend on action potentials and synaptic transmission. It may be that fMRI‐detected blood oxygenation level‐dependent signals (which are delayed relative to real‐time neuronal activity) remain after they are no longer reflective of current neural electric activity, or the changes they reflect are no longer sufficient to affect ongoing neural network activity. However, this possibility is challenged by the fact that the duration of a week far exceeds a delay between electric activity and blood oxygenation level‐dependent signals. Second, our CTRS‐TRD patient cohort may undergo rapid neurotransmitter desensitization, thereby weakening a synergistic ketamine effect. However, 1 week would be a short period of time for such desensitization. Moreover, if these patients exhibit rapid desensitization characteristics, it would be expected that desensitization would also affect their addiction tendency. Third, we postulate that DMN ReHo values may not be suitable indices of clinical effects given that ReHo data are derived from calculations rather than directly from microimaging. Thus, ReHo does not provide direct evidence for neural structural alterations or discharge activities.

A more notable phenomenon which should be addressed in additional secondary follow‐up studies is the observed inefficacy of the ketamine augmentation treatment regimen tested. The finding that ReHo alterations differed between our pilot and secondary follow‐up studies is also of great interest. While we are able to provide possible reasons for the dissociation between clinical and functional brain changes, and for rapid attenuation of ReHo by “desensitization inference,” we cannot account for the inefficacy of the ketamine augmentation treatment in our secondary follow‐up study. We can only postulate that ketamine may induce long‐term tolerance in patients with CTRS‐TRD. If this is true, then great vigilance must accompany administration of ketamine to patients with any type of mental disorder. However, if this is not the case, further study is needed, including animal studies, to investigate a possible reason for the observed “desensitization inference.”

### 4.1 Limitations

The pilot study we conducted had several limitations. First, since this line of research is an exploratory stage, information in the literature regarding the effects of low‐dose ketamine is limited. Moreover, the present evidence we collected is based on a small sample size since we only included patients with TRD and treatment‐resistant schizophrenia. While our evidence is not strong enough to influence clinical practice at this stage, it does provide valuable direction for further studies. Ideally, large cohort studies are needed to delineate and explain the effects of ketamine on depressive symptoms in schizophrenia. Second, the patients in our cohort were taking a variety of antidepressants, with most taking drugs from two different chemical constitution categories at the same time. Because we did not transfer antidepressant dosages to a uniform dosage, we cannot isolate the possible influence of antidepressants on our ReHo data. However, during our study, we did fix the dosage of all the therapeutic agents to reduce dynamic antidepressant influences on ReHo. Third, although ketamine has previously been reported to normalize aberrant functional connectivity (Abdallah, Jackowski, et al., 2017; Gartner et al., 2019), we observed that functional connectivity alterations did not withstand family‐wise error correction. This result may be due to our small sample size, which did not provide sufficient power. Fourth, when we calculated amplitudes of low‐frequency fluctuation before and after ketamine treatment (data not shown due to space limitations), we observed that regions of the brain which exhibited increased fluctuation amplitudes following ketamine administration overlapped to a large extent with brain regions exhibiting increased ReHo values. Furthermore, the latter increases did not correlate with symptom changes. Fifth, to better monitor ASEs, we only included patients with full insight, which excludes most schizophrenics. Thus, the generalizability of the current findings is potentially limited. Sixth, we only compared symptoms and changes in ReHo values before versus after ketamine treatment in a single group sample. Thus, although the strength of this study is not comparable to that of a randomized controlled trial, our findings provide important clues for future trials. Seventh, although most studies examining potential adverse effects of ketamine interactions with antidepressants have not found any (Duman & Shinohara, 2019), such effects were suggested in a recent study (Duman & Shinohara, 2019). In the present study, liver and renal function test results for our cohort were each within normal ranges. Because ketamine is metabolized by 3A4 and 2B6 enzymes, which are expressed primarily in the liver, potential metabolic ASEs are a serious concern. Eighth, although ketamine alone has been reported to alleviate TRD symptoms (Andrade, 2017), we did not ask our patients to stop taking their prescribed antidepressants in order to prevent a possible worsening of psychotic symptoms. Ninth, the secondary follow‐up study we conducted was based on the findings of our pilot study. Thus, validation of this method is needed, as well as further confirmation of our results. Tenth, prior to the secondary follow‐up study, the patients accepted rTMS treatment. Thus, it is possible that rTMS treatment may have influenced the findings of our follow‐up study, despite the treatment being discontinued prior to the start of our secondary follow‐up study. Eleventh, it is well established that ketamine can decrease functional activity in the DMN and other brain regions (Cheng et al., 2018; Hao et al., 2019; Ismaylova et al., 2018; Levenson et al., 2014; Nakamura et al., 2008; Qiu & Lin, 2019; Rodriguez et al., 2019; Schmaal et al., 2017; Smith et al., 2018; Soares et al., 2017; Subramaniam et al., 2018; Timbie & Barbas, 2015; van Eijndhoven et al., 2013; Zong et al., 2019). However, ReHo values were increased in the DMN of the CTRS‐TRD patients in our pilot study. We hypothesize that this seemingly contradictory finding may be related to neuropathological features of schizophrenia. Indeed, the neuropathological features of depressive symptoms in patients diagnosed with major depressive disorder have been reported to differ from those of depressive symptoms in schizophrenic patients (Jiang et al., 2017; Schilbach et al., 2016; Shao et al., 2018). Hence, we posit that ketamine‐induced ReHo alterations in schizophrenics may also differ from those in patients with major depressive disorder. Further research is needed to explain these findings. Twelfth, we observed that ReHo values were more widely increased in our secondary follow‐up study than in our pilot study. This is an unusual phenomenon and one that we reluctantly postulated to be related to an unstable feature of ReHo. It is important that further studies be conducted to more thoroughly investigate this phenomenon. Thirteenth, in this pilot study, we could not detail anhedonia, a pivotal symptom of depression and one of the symptoms which is observed in patients with substance abuse issues (Garfield et al., 2014; Upthegrove et al., 2017). Considering that ketamine has a potential addiction risk (although low dosages of ketamine generally do not induce an addiction risk), this does not mean that an addiction risk is excluded. Furthermore, we also observed rapid desensitization in our pilot study. Thus, alterations in anhedonia should be considered in patients undergoing ketamine augmentation treatment. However, because we lack an ideal tool for assessing alterations in anhedonia in patients with CTRS‐TRD, we did not detail this assessment in the present study. This is an obvious flaw in our study, and further studies are needed to clarify this event. Previous studies have reported that anhedonia is a common feature in both depression and negative symptoms of schizophrenia. However, consummatory anhedonia (pleasure experienced in better anticipation or in response to rewards) and difficulty in anticipating future pleasure may be more consistent with depression, whereas motivational anhedonia (motivation to pursue rewards) may be considered a primary negative symptom (Strauss & Gold, 2012; Upthegrove et al., 2010, 2017). Hence, further studies are needed to establish an ideal tool for assessing anhedonia symptoms in patients with CTRS‐TRD.

## 5 CONCLUSION

To the best of our knowledge, this pilot study and secondary follow‐up study are the first study to investigate the effects of adjunct ketamine treatment on TRD symptoms and concomitant functional brain alterations in patients with treatment‐resistant schizophrenia. Moreover, the present study represents a relatively long‐term study compared with previous studies. We observed that alleviation of depressive symptoms in CTRS‐TRD patients with adjunct ketamine treatment only lasted 1 week during the first treatment. Moreover, after 1 month, the antidepressant effect of ketamine had completely disappeared. Correspondingly, ReHo alterations induced by ketamine in our CTRS‐TRD patients were not maintained for more than 3 weeks. Thus, these findings indicate that adjunct ketamine treatment is unsatisfactory for CTRS‐TRD patients.

## CONFLICT OF INTEREST

The authors have no conflict of interest to declare.

## AUTHOR CONTRIBUTIONS

CZ, XL, and CC conceived and designed research, and revised the paper; CZ, XL, HT, and HB collected data and conducted research; CZ, XL, HT, SL, and CC analyzed and interpreted data; CZ wrote the initial paper; CZ had primary responsibility for final content. All authors read and approved the final manuscript.

## Data Availability

The datasets generated and analyzed during the present study are available from the corresponding author on reasonable request.
